# Transcriptome profiling of radiata pine branches reveals new insights into reaction wood formation with implications in plant gravitropism

**DOI:** 10.1186/1471-2164-14-768

**Published:** 2013-11-08

**Authors:** Xinguo Li, Xiaohui Yang, Harry X Wu

**Affiliations:** CSIRO Plant Industry, GPO Box 1600, Canberra, ACT 2601 Australia; Department of Biotechnology, Beijing Forestry University, Beijing, 100083 China; Umeå Plant Science Centre, Department of Forest Genetics and Plant Physiology, Swedish University of Agricultural Sciences, SE-901 83 Umeå, Sweden

**Keywords:** Compression wood, Tracheid, Conifers, Transcriptome, Microarray, Plant gravitropism, Microfibril angle (MFA), Wood stiffness

## Abstract

**Background:**

Formation of compression (CW) and opposite wood (OW) in branches and bent trunks is an adaptive feature of conifer trees in response to various displacement forces, such as gravity, wind, snow and artificial bending. Several previous studies have characterized tracheids, wood and gene transcription in artificially or naturally bent conifer trunks. These studies have provided molecular basis of reaction wood formation in response to bending forces and gravity stimulus. However, little is known about reaction wood formation and gene transcription in conifer branches under gravity stress. In this study SilviScan® technology was used to characterize tracheid and wood traits in radiate pine (*Pinus radiata* D. Don) branches and genes differentially transcribed in CW and OW were investigated using cDNA microarrays.

**Results:**

CW drastically differed from OW in tracheids and wood traits with increased growth, thicker tracheid walls, larger microfibril angle (MFA), higher density and lower stiffness. However, CW and OW tracheids had similar diameters in either radial or tangential direction. Thus, gravity stress largely influenced wood growth, secondary wall deposition, cellulose microfibril orientation and wood properties, but had little impact on primary wall expansion. Microarray gene transcription revealed about 29% of the xylem transcriptomes were significantly altered in CW and OW sampled in both spring and autumn, providing molecular evidence for the drastic variation in tracheid and wood traits. Genes involved in cell division, cellulose biosynthesis, lignin deposition, and microtubules were mostly up-regulated in CW, conferring its greater growth, thicker tracheid walls, higher density, larger MFA and lower stiffness. However, genes with roles in cell expansion and primary wall formation were differentially transcribed in CW and OW, respectively, implicating their similar diameters of tracheid walls and different tracheid lengths. Interestingly, many genes related to hormone and calcium signalling as well as various environmental stresses were exclusively up-regulated in CW, providing important clues for earlier molecular signatures of reaction wood formation under gravity stimulus.

**Conclusions:**

The first comprehensive investigation of tracheid characteristics, wood properties and gene transcription in branches of a conifer species revealed more accurate and new insights into reaction wood formation in response to gravity stress. The identified differentially transcribed genes with diverse functions conferred or implicated drastic CW and OW variation observed in radiata pine branches. These genes are excellent candidates for further researches on the molecular mechanisms of reaction wood formation with a view to plant gravitropism.

**Electronic supplementary material:**

The online version of this article (doi:10.1186/1471-2164-14-768) contains supplementary material, which is available to authorized users.

## Background

Trees can change their growth orientation in response to various environmental stresses (ie, wind, snow, light, gravity, artificial bending) [1], and during this process reaction wood with modified characteristics and properties is formed. In gymnosperms, compression wood (CW) is produced on the lower side of inclined (bent) trunks or naturally growing branches [2]. This reaction wood helps conifer trees maintain stem straightness and branches at certain angles. Characterization of CW formed in bent trunks has been extensively investigated in many conifer species [1, 3–10]. Compared to its opposite wood (OW) formed on the upper side of branches and bent trunks, CW has shorter tracheids, larger microfibril angle (MFA), greater shrinkage, higher density, lower stiffness, more lignin and less cellulose [1, 3–10]. Thus, it is considered undesirable for both solid wood and pulp products [10, 11].

Molecular basis of CW formation has been previously studied in the bent trunks of several conifer species. Using the semi-quantitative RT-PCR technique a few cell wall structural protein genes and several lignin-related genes were identified with differential transcription in the bent trunks of loblolly pine [12] and Japanese cypress [9, 13], respectively. Based on suppression subtractive hybridization (SSH) and qRT-PCR, several genes involved in cell wall modification, lignin biosynthesis and transcription regulation had differential transcription in the inclined stems of radiata pine and maritime pine [14]. Furthermore, genomic approaches such as cDNA microarrays revealed differential gene transcription in the bent trunks of loblolly pine [15] and maritime pine [16]. These studies have provided valuable insights into the molecular basis of CW formed in bunt trunks of conifers, particularly with regard to its higher lignin content.

To our knowledge all published researches on the properties and gene transcription of reaction wood in conifer species (CW) and angiosperms (tension wood, TW) were based on the bent trunks under artificial bending or natural inclining forces. It should be noted that these trees had experienced both external forces and gravity stimulus. Particularly, the bending force in young trees and seedlings could be much larger than gravity stress. Thus, wood properties and gene transcription observed in bent trunks may not accurately reflect gravity stress. In contrast, naturally grown branches are mostly under gravity stress, providing excellent materials for the study of wood formation in response to gravity stimulus. Although one previous study with eucalyptus branches identified a few cell wall genes in response to gravity stress in angiosperms [17], wood property variation and the xylem transcriptome changes between the upper and lower sides of branches remains largely unknown in any conifer species.

Radiata pine (*Pinus radiata* Don.) is the most important conifer species for commercial forestry in Australia, New Zealand and Chile. CW properties of radiata pine have been previously characterized using inclined trees [3, 6, 8]. In the present study radiata pine branches were used to study CW formation. Firstly, tracheid characteristics and wood properties were measured using the SilviScan® technology [18, 19]. Then, differential gene transcription between the upper (OW) and lower sides of branches (CW) was investigated using radiata pine cDNA microarrays. The aim of this study is to reveal insights into the molecular mechanisms of reaction wood formation in conifer branches with a view to plant gravitropism.

## Results

### Characterization of tracheid and wood traits in CW and OW of branches

In the cross-section of the six branch discs, average radius of the lower side xylem (CW) was 4.6 cm, significantly longer than that of total OW formed on the upper side of branches (3.1 cm, P-value = 0.0002). The six branch discs had 10 growth rings in the cross-section. Within a ring formed in a growing circle CW was significant wider than OW (P-values ≤ 0.01) except for the first four rings from pith. These results indicated that gravity stress significantly increased wood formation on the lower side of branches. The larger wood growth in CW could generate compression force to maintain branches at certain orientation.

**Figure 1:**
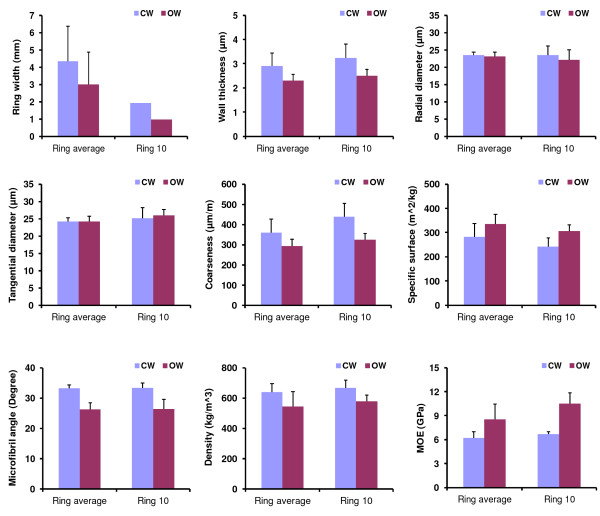
**Variation in tracheid characteristics and wood properties between compression (CW) and opposite wood (OW) of branches.** Ring width, tracheid wall thickness, radial diameter, tangential diameter, coarseness, specific surface, microfibril angle (MFA), wood density and stiffness (modulus of elasticity, MOE) were measured in six wood strips of radiata pine branches using SilviScan 2. Average ring values of each trait were compared between the lower side (CW) and upper side (OW) of the six branches. Tracheid and wood traits in ring 10 representing developing xylem tissues collected for microarray experiments were also compared between CW and OW. Error bars represent the standard deviation of the mean value of each trait. CW and OW variation is statistically significant (P-values ≤ 0.05) except for the two tracheid diameters.

SilviScan measurement of the six branch discs showed significant differences between CW and OW in wood growth, tracheid characteristics and wood properties in terms of average ring values (Figure 1). Significant CW and OW variation was also observed in ring 10 (Figure 1), which represented developing xylem tissues sampled for the two microarray experiments. In both comparisons CW had greater growth, thicker tracheid walls, larger MFA, greater coarseness, lower specific surface, higher density and lower stiffness compared to OW. Interestingly, MFA was drastically altered during CW formation. Average MFA in CW (lower side) of branches was 33.2 degrees, significantly larger than that of OW (26.2 degrees). Surprisingly, diameters of CW tracheids were similar to that of OW in both radial and tangential directions, respectively (Figure 1). Thus, gravity stress appeared to have little influence on tracheid dimensions in the two directions. Moreover, radial dimension of CW and OW tracheids (24.2-24.3 μm) was slightly larger than their tangential dimension (23.1-23.5 μm), resulting in nearly round or square shapes of tracheids in the cross-section.

### Transcriptome comparison between CW and OW formed in branches

**Figure 2:**
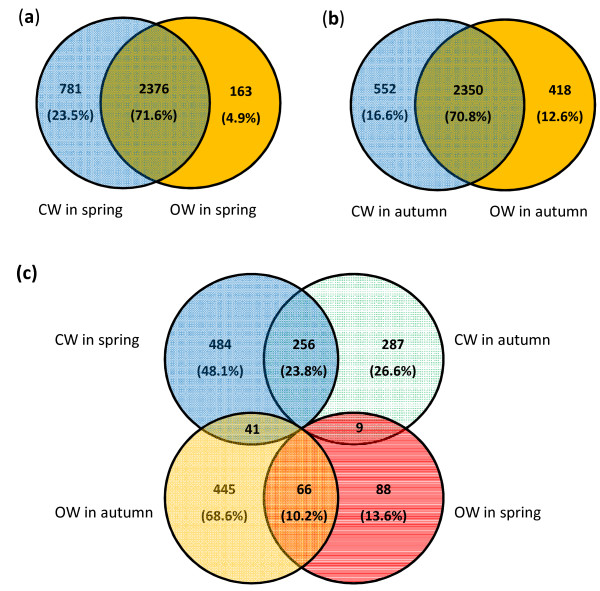
**Transcriptome comparisons between compression (CW) and opposite wood formed in branches.** Genes differentially transcribed in CW and OW sampled in spring and autumn were identified using radiata pine cDNA microarrays, respectively. Numbers of preferentially transcribed genes identified from developing xylem sampled in spring **(a)** and autumn **(b)** were present. Differentially transcribed genes were further compared between the two seasons. A number of genes showed consistently differential transcription in the two wood tissues across the two seasons **(c)**.

The xylem transcriptomes of CW and OW sampled in spring (called earlywood, EW) and autumn (latewood, LW) were compared respectively using radiata pine cDNA microarrays. In the first comparison between CW and OW sampled in spring, 944 out of 3,320 xylem unigenes (28.4%) on the microarrays had differential transcription, including 781 and 163 unigenes preferentially transcribed in CW and OW, respectively (Figure 2a). Using samples collected in autumn slightly more unigenes (970, 29.2%) were identified with differential transcription (552 and 418 unigenes for CW and OW, respectively) (Figure 2b). Thus, different growing seasons may only have little impact on the proportion of the xylem transcriptomes differentially transcribed in CW and OW of radiata pine branches. However, genes up-regulated in CW in spring (781) were five times more than that in OW (163); while in autumn genes preferentially transcribed in CW were slightly more than that in OW. Nearly half of the identified genes (46.4% for CW and 40.5% for OW) had similar transcription patterns in the two seasons during reaction wood formation (Figure 2c).

The two microarray experiments identified a total of 1,204 and 514 genes differentially transcribed in CW and OW, respectively (Additional file 1). Almost all these genes (98.0% for CW and 98.6% for OW) had close matches in the UniProt known proteins and TIGR gene indices databases (tblastx, E-value ≤ 1e-5). However, about 35% of the matches did not have a clear function. This is because CW and OW formation has been poorly characterized at the molecular level. Of 1,204 genes identified in CW, 588 genes were annotated with gene ontology (GO) terms, and majority (90.6%) showed molecular functions, 75.5% had roles in biological processes and less than half (48.6%) might be cellular components. Similarly, in the 514 genes preferentially transcribed in OW 276 transcripts were annotated with GO terms, including molecular functions (85.5%), biological process (74.3%) and cellular components (47.8%).

**Figure 3:**
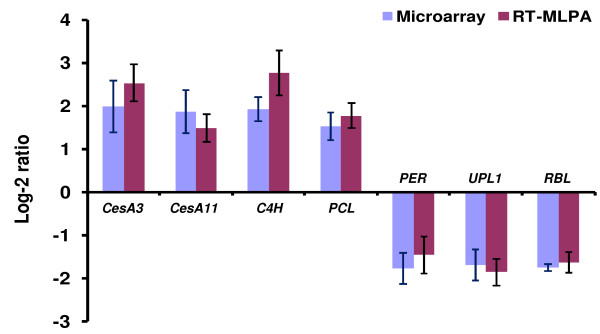
**Validation of microarray transcription of selected differentially transcribed genes.** A total of seven differentially transcribed genes were selected in the validation using reverse transcriptase-multiplex ligation dependent probe amplification (RT-MLPA). These genes include four genes up-regulated in CW: *cellulose synthase 3* (*PrCesA3*), *PrCesA11*, *cinnamic acid 4-hydroxylase* (*C4H*) and *plastocyanin-like* (*PCL*); three genes more highly transcribed in OW: *peroxidase* (*PER*), *E3 ubiquitin protein ligase* (*UPL1*) and *retinoblastoma-like protein* (*RBL*). Developing xylem (CW and OW) sampled in autumn for the microarray experiment was used in the validation, including three biological and four technical replicates. Mean log-2 ratios (CW/OW) of the 12 replicates were calculated for the selected genes and compared with their microarray transcription results. The mean log-2 ratio values > 0 and < 0 indicate genes preferentially transcribed in CW and OW, respectively. Error bars represent the standard deviation of the mean log-2 ratio.

Microarray results of seven selected genes with differential transcription in CW and OW sampled in autumn were validated using the RT-MLPA method. The magnitudes of differential gene transcription measured by RT-MLPA had no significant differences compared to that in the microarray experiment (P-values ≤ 0.05) (Figure 3). This result indicated that the microarray experiments conducted in this study were sufficiently reliable for the identification of genes differentially transcribed in lower and upper sides of radiata pine branches under gravity stress.

### Differential transcription of cytoskeleton-related genes

From a total of 1,728 genes with differential transcription identified in this study, 28 genes were involved in actin filaments and microtubules (Table 1). In the development of actin filaments, genes encoding different actins, actin bundling proteins and actin related proteins were preferentially transcribed in either CW or OW. However, *actin depolymerizing factor* (*ADF*) and *ADF-like* genes were exclusively up-regulated in CW of branches. In the microtubule development, different members of the same gene families encoding tubulin folding cofactors, microtubule-associated proteins (MAPs) and MAP kinases were differentially transcribed in CW and OW, respectively. Interestingly, seven *tubulins* (two *alpha*- and five *beta-tubulins*) were exclusively up-regulated in CW sampled in the two seasons (three *tubulins*) or a single season (four).

**Table 1:** **Cytoskeleton-related genes differentially transcribed in compression (CW) and opposite wood (OW) formed in branches**

***Xylem tissues***	Differentially transcribed genes*	***Representing ESTs***	***GenBank accession***	***Log-2 ratios***	***P-values***
CW-spring	*Actin*	TWL22A2	FE523923	1.532	0.048
CW-spring	*Actin 3*	MWL23B3	GO269503	0.905	0.003
CW-autumn	*Actin 3*	JWLxF8	FE521083	0.574	0.033
CW-autumn	*Actin 7*	JWEeC7	FE518833	2.150	0.048
CW-spring	*Actin 7*	TWE23D12	FE523186	1.794	0.050
CW-spring	*Actin bundling protein ABP135*	TWE5D1	FE523228	0.800	0.010
CW-autumn	*Actin depolymerizing factor*	MWE24D11	FE521861	2.167	0.034
CW-spring	*Actin depolymerizing factor*	MWE5G10	FE521693	1.772	0.047
CW-autumn	*Actin depolymerizing factor-like*	JWEqB11	FE518555	0.664	0.011
CW-spring	*Actin filament bundling protein P-115*	JWL19E9	FE521315	1.676	0.048
CW-autumn	*Actin filament bundling protein P-115*	JWL19E9	FE521315	1.539	0.016
CW-autumn	*Actin related protein 2*	JWLfF5	FE519755	1.159	0.035
CW-autumn	*Actin related protein 3*	JWEmB5	FE518964	1.211	0.040
CW-autumn	*Tubulin alpha*	JWEbH8	FE519649	1.942	0.049
CW-spring	*Tubulin alpha 1*	JWEsG6	FE519115	1.580	0.033
CW-autumn	*Tubulin alpha 1*	TWL9D10	FE524256	1.830	0.050
CW-spring	*Tubulin beta*	JWLlA9	FE519966	1.944	0.037
CW-autumn	*Tubulin beta 1*	JWLwC2	FE520255	1.694	0.044
CW-spring	*Tubulin beta 1*	JWLwC2	FE520255	1.706	0.010
CW-spring	*Tubulin beta 3*	MWE31B9	FE521866	1.571	0.042
CW-spring	*Tubulin beta 6*	MWL30D8	FE522772	1.340	0.047
CW-autumn	*Tubulin beta 6*	JWLrC8	FE520019	1.557	0.032
CW-spring	*Tubulin beta 7*	JWLhG4	FE520687	1.993	0.024
CW-spring	*Tubulin folding cofactor B*	TWL22B11	FE524340	2.023	0.050
CW-autumn	*Microtubial binding protein*	MWE30H10	FE521906	0.650	0.026
CW-autumn	*MAP-like*	MWE9A10	FE521409	1.479	0.009
CW-spring	*MAP kinase-like*	MWE31C11	FE521885	1.888	0.015
OW-spring	*Actin 2*	TWE16C11	FE523366	-1.470	0.049
OW-autumn	*Actin 2*	MWL29C3	FE522532	-2.139	0.049
OW-autumn	*Actin bundling protein ABP135*	TWE5D1	FE523228	-1.162	0.019
OW-autumn	*Actin related protein 6*	TWE4H5	FE523100	-2.163	0.042
OW-autumn	*Tubulin folding cofactor A*	MWL33E1	FE522629	-1.448	0.000
OW-spring	*MAP*	JWLlA3	FE520828	-1.777	0.043
OW-autumn	*MAP*	JWL11A8	FE521181	-1.328	0.043
OW-autumn	*MAP kinase*	JWEiD6	FE518418	-1.342	0.010
OW-autumn	*MAP kinase phosphatase*	MWE1B4	FE521481	-1.621	0.007
OW-spring	*MAPKK*	TWE5D10	FE523280	-1.982	0.014
OW-autumn	*MAPKK*	TWE5D10	FE523280	-1.914	0.034

*MAP, microtubule-associated protein; MAPKK, mitogen-activated protein kinase kinase.

### Differential transcription of cell wall-related genes

Genes related to cell wall formation at various developmental stages were identified with differential transcription in CW and OW of radiata pine branches. Interestingly, these genes were more frequently up-regulated in CW than in OW (Table 2 and Table 3). In cell division, four genes (*cell cycle switch protein*, *cell division cycle protein 48*, *cyclin*, *profilin-1*) were up-regulated in CW; while only one gene (*cyclin-like F-box domain*) was preferentially transcribed in OW. In cell expansion, three *expansin* and *extensin* genes were up-regulated in CW (*expansin beta 1*, *expansin ripening related* and *extensin-like*); while only one up-regulated in OW (*expansin alpha 3*). In pectin biosynthesis, three genes (*pectin lyase 2*, *pectinesterase-like* and *pectin-glucuronyltransferase*) were up-regulated in OW; while only a *pectin lyase-like* had higher transcription in CW. In primary wall modification, two *xyloglucan endotransglucosylase/hydrolases* (*XET8* and *32*) were up-regulated in CW of branches, and a gene encoding ovule/fiber cell elongation proteins was more highly transcribed in OW.

**Table 2:** **Cell division and primary wall modification genes differentially transcribed in compression (CW) and opposite wood (OW) formed in branches**

***Xylem tissues***	Differentially transcribed genes*	***Representing ESTs***	***GenBank accession***	***Log-2 ratios***	***P-values***
CW-spring	*Cell cycle switch protein*	MWL10H5	FE522899	2.570	0.048
CW-autumn	*Cell cycle switch protein*	MWL10H5	FE522899	0.893	0.041
CW-spring	*Cell division cycle protein 48*	TWE20C6	FE523511	1.064	0.041
CW-spring	*Cyclin*	JWL24C10	FE520640	2.214	0.031
CW-spring	*Profilin-1*	MWE1E2	FE521468	1.310	0.037
CW-autumn	*Profilin-1*	MWE6H12	FE521797	2.001	0.040
CW-spring	*Expansin-B1 precursor*	JWLsE7	FE520098	2.584	0.037
CW-autumn	*Expansin-B1 precursor*	JWLsE7	FE520098	1.527	0.041
CW-spring	*Expansin, Ripening-related*	JWLlF11	FE520876	1.973	0.040
CW-autumn	*Expansin, Ripening-related*	MWL18G4	FE522961	1.753	0.040
CW-spring	*Extensin-like protein*	JWEuG12	FE519300	1.831	0.003
CW-autumn	*XET8*	JWEqD8	FE519429	1.656	0.047
CW-autumn	*XET32*	JWLvE1	FE520165	0.976	0.017
CW-spring	*Pectate lyase-like*	MWL25G12	FE522408	1.347	0.047
CW-spring	*Cellulase 24*	MWL15E11	FE522326	1.455	0.036
OW-autumn	*Cyclin-like F-box*	JWL11H8	FE520391	-2.085	0.034
OW-spring	*Expansin alpha- 3*	TWE13E12	FE523142	-1.650	0.016
OW-autumn	*Expansin alpha- 3*	TWE33F8	FE523674	-2.028	0.042
OW-spring	*Pectate lyase 2*	TWE8C12	FE523428	-1.453	0.019
OW-autumn	*Pectate lyase 2*	TWE8C12	FE523428	-1.674	0.046
OW-spring	*Pectinesterase-like*	JWLjC8	FE521362	-1.460	0.032
OW-autumn	*Pectin-glucuronyltransferase*	TWE16C7	FE523338	-1.898	0.042
OW-autumn	*Ovule/fiber cell elongation protein*	TWE15H7	GO269508	-0.628	0.049

*XET, xyloglucan endotransglucosylase/hydrolase.

**Table 3:** **Genes related to cellulose and lignin biosynthesis differentially transcribed in compression (CW) and opposite wood (OW) formed in branches**

***Xylem tissues***	Differentially transcribed genes*	***Representing ESTs***	***GenBank accession***	***Log-2 ratios***	***P-values***
CW-spring	*PrCesA3*	TWL7C2	FE524088	1.316	0.045
CW-autumn	*PrCesA3*	TWL1D5	FE524207	1.984	0.046
CW-autumn	*PrCesA7*	JWE3D1	FE518578	1.464	0.041
CW-spring	*PrCesA11*	TWL21B5	FE523881	1.336	0.041
CW-autumn	*PrCesA11*	TWL28A3	FE523830	1.867	0.038
CW-spring	*PrCesA-like*	JWLvB12	FE520241	1.661	0.016
CW-autumn	*PrCesA-like*	JWLvB12	FE520241	1.417	0.012
CW-spring	*Sucrose synthase*	TWL8B9	FE524247	1.467	0.038
CW-autumn	*Sucrose synthase*	JWEtG3	FE519171	2.153	0.045
CW-spring	*Sucrose synthase 1*	JWE5H8	FE519487	1.377	0.033
CW-spring	*Callose synthase-like*	TWL14D7	FE524183	0.933	0.028
CW-spring	*Glycosyl transferase 48*	JWLxB9	FE521086	1.444	0.037
CW-spring	*Chorismate synthase*	TWL12H4	FE524274	2.155	0.046
CW-autumn	*Chorismate synthase*	TWL12H4	FE524274	1.336	0.177
CW-spring	*Chorismate mutase*	MWE4C8	FE521447	1.308	0.010
CW-spring	*Shikimate kinase 2*	JWL17G12	FE520492	2.685	0.046
CW-spring	*EPSPS*	JWLbD8	FE519700	2.111	0.018
CW-autumn	*EPSPS*	JWLbD8	FE519700	1.906	0.040
CW-spring	*DAHPS*	MWE11H6	FE521953	1.295	0.048
CW-autumn	*DAHPS*	MWE11H6	FE521953	1.966	0.041
CW-spring	*4CL*	JWE8F8	FE519348	1.289	0.046
CW-autumn	*4CL*	JWEnE8	FE519597	1.830	0.046
CW-spring	*C4H*	TWL23G12	FE523933	1.547	0.047
CW-autumn	*C4H*	MWE8E8	FE522027	1.921	0.048
CW-spring	*CCR-like*	JWE3H12	FE518650	1.180	0.043
CW-spring	*COMT*	MWL29C12	FE522592	1.449	0.049
CW-autumn	*COMT*	MWE7C3	FE522592	1.571	0.014
CW-autumn	*PAL2*	JWL4F3	FE521295	1.635	0.050
CW-spring	*Laccase 2*	JWL22G2	FE520504	1.810	0.049
CW-autumn	*Laccase 2*	JWL22G2	FE520504	1.238	0.039
CW-spring	*Laccase 4*	TWL13F12	FE524173	1.616	0.070
CW-autumn	*Methionine synthase*	JWLuE3	FE520972	1.558	0.031
CW-autumn	*Methionine synthase 2*	JWEuE1	FE519234	1.663	0.049
CW-autumn	*MetE*	MWE9E1	GO269413	1.749	0.045
CW-spring	*SAMS*	TWL18G9	FE523865	1.552	0.044
CW-autumn	*SAMDC*	JWE8E2	FE519501	1.881	0.048
CW-spring	*Dirigent-like protein pDIR3*	JWLf10D9	FE519781	1.493	0.035
CW-autumn	*Dirigent-like protein pDIR3*	TWL26F6	FE524371	1.226	0.043
CW-autumn	*Dirigent-like protein pDIR4*	JWLwD4	FE520269	1.807	0.042
CW-autumn	*Dirigent-like protein pDIR14*	JWEdE1	FE518247	2.298	0.042
OW-autumn	*PrCesA10*	TWE21G7	FE523538	-2.497	0.041
OW-autumn	*Glycosyl transferase 8-like*	TWE16F7	FE523341	-1.532	0.048
OW-autumn	*Glycosyl transferase NTGT5a*	TWE13G1	FE523678	-1.638	0.047
OW-spring	*COMT-like*	JWLkA8	FE520785	-2.112	0.035
OW-spring	*Peroxidase precursor*	TWE24D10	FE523740	-1.376	0.030
OW-autumn	*Peroxidase precursor*	TWE27F5	FE523746	-1.774	0.032
OW-spring	*Peroxidase PSYP1,Class III*	MWL21F8	GO269502	-1.999	0.039
OW-autumn	*Peroxidase PSYP1,Class III*	MWL21F8	GO269502	-1.474	0.045
OW-autumn	*Dirigent protein pDIR18*	TWE21H12	FE523546	-0.636	0.021

*PrCesA, Pinus radiata cellulose synthase; *4CL, 4-coumarate:CoA ligase*; *C4H, cinnamic acid 4-hydroxylase*; *CCR, cinnamoyl CoA reductase*; *COMT, caffeic acid ortho-methyltransferase*; *PAL, phenylalanine ammonia-lyase*; *DAHPS*, *3-deoxy-D-arabino-heptulosonate 7-phosphate synthase*; *EPSPS, 5-enolpyruvylshikimate 3-phosphate synthase*; *SAMS, S-adenosylmethionine synthetase*; *SAMDC*, *S-adenosylmethionine decarboxylase*; *MetE, cobalamin-independent methionine synthase*.

Secondary cell wall genes were mostly up-regulated in CW of radiata pine branches (Table 3). Of genes involved in cellulose biosynthesis, *cellulose synthases* (*PrCesA3*, *7*, *11*), *PrCesA-like* and *sucrose synthases* (*SuSy* and *SuSy1*) were preferentially transcribed in CW. Four of these genes (*PrCesA3*, *11*, *PrCesA-like* and *SuSy*) were up-regulated in CW sampled in both spring and autumn. In the lignin-related genes, *4-coumarate:CoA ligase* (*4CL*), *cinnamic acid 4-hydroxylase* (*C4H*), *cinnamoyl CoA reductase-like* (*CCR-like*), *caffeic acid ortho-methyltransferase* (*COMT*), *phenylalanine ammonia-lyase 2* (*PAL2*), *methionine synthases*, *S-adenosylmethionine synthetase* (*SAMS*), *S-adenosylmethionine decarboxylase* (*SAMDC*)*, laccases* (*LAC2* and *4*) and *dirigent-like* were up-regulated in CW. A wide range of cell wall structural protein genes were up-regulated in CW, such as *arabinogalactan proteins* (*AGP4*, *5*, *6* and *AGP-like*), *fasciclin-like arabinogalactan proteins* (*FLA1* and *8*), *glycine-rich protein*, *proline-rich protein* and so on (Table 4). In contrast, only three *FLAs* (*FLA10*, *17* and *26*) were preferentially transcribed in OW of branches.

**Table 4:** **Cell wall structural protein genes differentially transcribed in compression (CW) and opposite wood (OW) formed in branches**

***Xylem tissues***	Differentially transcribed genes*	***Representing ESTs***	***GenBank accession***	***Log-2 ratios***	***P-values***
CW-spring	*Arabinogalactan/proline-rich protein, AGP4*	MWE13B9	FE521826	1.559	0.050
CW-spring	*Arabinogalactan protein 5, AGP5*	TWL29C3	FE524468	1.593	0.037
CW-autumn	*Arabinogalactan protein 5, AGP5*	TWL1A1	FE524552	2.018	0.049
CW-spring	*Arabinogalactan protein 6, AGP6*	JWEmA4	FE518957	1.330	0.038
CW-autumn	*Arabinogalactan protein 6, AGP6*	JWLtE9	FE520147	2.009	0.035
CW-spring	*Arabinogalactan-like*	MWE12B5	FE518750	1.826	0.031
CW-autumn	*Arabinogalactan-like*	MWE12B5	FE518750	1.466	0.046
CW-spring	*FLA1*	MWL28F5	FE522423	0.421	0.004
CW-spring	*FLA8*	JWLxF4	FE521059	2.546	0.029
CW-autumn	*FLA8*	JWLxF4	FE521059	1.911	0.037
CW-spring	*Glycine-rich protein 1*	MWE6G10	FE521816	2.044	0.017
CW-autumn	*Glycine-rich protein 1*	MWE6G10	FE521816	1.469	0.016
CW-spring	*Glycine-rich protein 2*	JWEgF6	FE519572	1.577	0.039
CW-autumn	*Glycine-rich protein 2*	JWErH8	FE519061	1.519	0.031
CW-spring	*Proline-rich protein*	MWE5D11	FE521995	1.521	0.047
CW-spring	*Plastocyanin-like*	TWL16D4	FE523902	1.464	0.044
CW-autumn	*Plastocyanin-like*	TWL26B4	FE524367	1.525	0.049
CW-autumn	*Plantacyanin*	TWL22G11	GO269524	1.513	0.043
CW-spring	*Uclacyanin 3*	TWL19C11	FE524322	1.104	0.020
CW-autumn	*Uclacyanin 3*	TWL19C11	FE524322	1.433	0.028
CW-spring	*Blue copper protein*	JWLbE4	FE519677	1.944	0.042
CW-autumn	*Blue copper protein*	TWL19E3	GO269525	2.235	0.047
OW-autumn	*FLA10*	MWE16A4	FE522128	-1.654	0.003
OW-autumn	*FLA17*	TWL29A10	FE524499	-1.736	0.050
OW-autumn	*FLA26*	TWE27C11	FE523610	-2.270	0.049

*FLA, fasciclin-like arabinogalactan protein.

### Hormone and calcium signalling genes differentially transcribed in CW and OW formation

Similar to genes involved in cell wall formation, differentially transcribed genes related to hormone and calcium signalling were mostly up-regulated in CW of branches; while only a few of these genes were preferentially transcribed in OW (Table 5). These hormone signalling genes were related to five major hormones (auxin, gibberellin, cytokinin, ethylene and abscisic acid). For example, three auxin-related genes were up-regulated in CW, including *auxin-induced protein*, *auxin-regulated protein* (*ARP*) and *ARP-like*. Other hormone-related genes up-regulated in CW included genes encoding gibberellins-induced receptor-like kinase, cytokinin-binding protein, ethylene response factor-like, ethylene responsive element binding factor and ethylene-forming enzyme. In addition, six calcium-related genes (*calcium dependent protein kinase*, *calcium/calmodulin-dependent protein kinase CaMK3*, *calcium-binding EF-hand*, *calcium-binding protein*, and *calcium-binding protein-like*) were up-regulated in CW of branches in both spring and autumn (Table 5).

**Table 5:** **Genes related to hormone and calcium signalling were differentially transcribed in compression (CW) and opposite wood (OW) formed in branches**

***Xylem tissues***	***Differentially transcribed genes***	***Representing ESTs***	***GenBank accession***	***Log-2 ratios***	***P-values***
CW-autumn	*Auxin-induced protein*	JWLwB8	FE520292	1.587	0.043
CW-spring	*Auxin-regulated protein*	JWElD4	FE519586	1.206	0.026
CW-spring	*Auxin-regulated protein-like*	MWL10D2	FE522886	1.964	0.049
CW-autumn	*Auxin-regulated protein-like*	MWL5D1	FE522833	2.045	0.044
CW-autumn	*Cytokinin-binding protein*	JWLuD5	FE520986	1.839	0.049
CW-spring	*Ethylene reponse factor-like*	MWL23D7	FE522376	2.893	0.051
CW-spring	*Ethylene responsive element binding factor*	JWEmG12	FE519013	1.103	0.032
CW-spring	*Ethylene-forming enzyme*	JWEhA9	FE518368	1.392	0.038
CW-autumn	*Ethylene-forming enzyme*	JWEhC9	FE518368	1.514	0.041
CW-autumn	*Gibberellin-induced receptor-like kinase*	TWL24D10	FE523925	1.959	0.006
CW-spring	*Abscisic acid-induced protein*	TWE30F7	FE523634	0.983	0.021
CW-spring	*Calcium dependent protein kinase*	JWLbH5	FE519684	2.196	0.049
CW-autumn	*Calcium dependent protein kinase*	JWLbH5	FE519684	1.691	0.050
CW-spring	*Calcium/calmodulin-dependent protein kinase*	TWL20F6	FE523844	1.832	0.005
CW-autumn	*Calcium/calmodulin-dependent protein kinase*	TWL20F6	FE523844	1.660	0.039
CW-spring	*Calcium-binding EF-hand*	JWLxA12	FE521106	1.077	0.018
CW-autumn	*Calcium-binding EF-hand*	JWLrA7	FE520012	2.045	0.048
CW-spring	*Calcium-binding protein*	MWL14A2	FE523065	0.086	0.036
CW-autumn	*Calcium-binding protein*	MWL14A2	FE523065	1.853	0.067
CW-spring	*Calcium-binding protein-lik*	MWL21C7	FE522357	1.053	0.0002
CW-autumn	*Calcium-binding protein-lik*	MWL21C7	FE522357	1.248	0.016
OW-autumn	*Abscisic acid-induced protein*	TWE30F7	FE523634	-0.912	0.033
OW-autumn	*Calcium-transporting ATPase 2*	MWL33C2	FE522634	-1.896	0.048

### CW and OW formation involves extensive transcription regulation

Most hormone signalling genes up-regulated in CW (Table 5) had functions in transcription regulation. Besides, many other transcription factor (TF) genes were also identified with differential transcription in CW and OW (Additional files 2). Different members of *homeodomain*, *LIM* and *Zinc finger* gene families were up-regulated in CW and OW, respectively. In contrast, several other TFs were preferentially transcribed in either CW or OW formation. For example, *BHLH*, *BTF*, *HD-ZIP* and *MYB* were exclusively up-regulated in CW; while *WRKY* and *transcriptional corepressor* were only highly transcribed in OW.

### Differential gene transcription related to divergent environmental stresses

Fifteen genes involved in various environmental stresses (i.e., water, light, diseases and salt) were up-regulated in CW of branches; while only two genes related to environmental stresses were preferentially transcribed in OW (Table 6). Of genes related to water stress, *aquaporin*, *water deficit inducible protein*, *dehydrin*, *dehydrin 1* and *dehydration-responsive protein-like* were up-regulated in CW. Several genes responding to salt stress (*salt tolerance protein 1*, *2* and *salt-induced AAA-type ATPase*) and disease resistance (*disease resistance gene*, *nucleotide-binding site (NBS) protein* and *TIR/P-loop/LRR*) were exclusively up-regulated in CW formation. Surprisingly, *light-inducible protein ATLS1*, *light-induced protein-like* and *phytochrome* were exclusively up-regulated in CW, suggesting CW formation may be affected by light signals.

**Table 6:** **Genes responding to environmental stresses differentially transcribed in compression (CW) and opposite wood (OW) formed in branches**

***Xylem tissues***	***Differentially transcribed genes***	***Representing ESTs***	***GenBank accession***	***Log-2 ratios***	***P-values***
CW-autumn	*Aquaporin*	MWL5E11	FE522873	2.002	0.045
CW-spring	*Water deficit inducible protein*	TWE12B12	FE523997	2.102	0.008
CW-autumn	*Water deficit inducible protein*	TWE12B12	FE523997	1.059	0.008
CW-spring	*Dehydrin*	TWL14B4	FE524283	1.415	0.036
CW-autumn	*Dehydrin*	TWL36E7	FE524023	2.125	0.045
CW-spring	*Dehydrin 1*	JWLwA1	FE520245	2.175	0.019
CW-spring	*Dehydration-responsive protein-like*	TWL17D5	FE523858	1.534	0.038
CW-spring	*Light-inducible protein ATLS1*	MWE5D1	FE521627	1.785	0.039
CW-autumn	*Light-inducible protein ATLS1*	TWL27A8	GO269536	1.648	0.039
CW-spring	*Light-induced protein like*	JWEcC9	FE518768	0.932	0.051
CW-spring	*Phytochrome*	TWL37H8	FE524199	1.347	0.044
CW-autumn	*Phytochrome*	TWL5H7	FE524072	1.945	0.046
CW-spring	*Disease resistance gene*	JWLlA4	FE520833	1.733	0.043
CW-autumn	*Disease resistance gene*	TWL36H4	FE523993	1.454	0.044
CW-autumn	*nucleotide-binding site (NBS) protein*	JWLjF7	FE519913	0.969	0.041
CW-spring	*TIR/P-loop/LRR*	TWL27G11	FE524445	1.159	0.032
CW-spring	*Multidrug resistance associated protein 1*	JWLwF8	FE520296	1.350	0.034
CW-spring	*Multidrug resistance associated protein 6*	MWE4G8	FE521986	1.228	0.002
CW-spring	*Salt tolerance protein 1*	JWLxA5	FE521062	1.569	0.047
CW-autumn	*Salt tolerance protein 1*	JWLxA5	FE521062	1.515	0.039
CW-spring	*Salt tolerance protein 4*	MWE29C5	FE521898	1.798	0.048
CW-autumn	*Salt-induced AAA-Type ATPase*	JWE1D5	FE519463	0.998	0.024
OW-autumn	*NBS/LRR*	MWL21G9	FE523021	-1.441	0.042
OW-spring	*Aluminium induced protein*	TWL29A2	FE524459	-1.615	0.044

## Discussion

### Extensive transcriptome remodelling underlies drastic CW and OW variation

Drastic variation between CW and OW of radiata pine branches indicated that gravity stimulus affects cell division, secondary wall deposition, cellulose microfibril orientation and overall wood properties. The larger MFA in CW greatly contributes to its lower wood stiffness despite of its higher density. This is because MFA rather than density has a predominant and adverse effect on wood stiffness [20]. The greater growth of CW on the lower side branches helps to push the branches up; while the lower MFA and higher stiffness in OW could contribute to pull up branches against gravitational force. Since TW formed on the upper side of angiosperm branches also had a drastically declined MFA and larger stiffness [17, 21], this pull-push mechanism appears to be conserved in gymnosperms and angiosperms. CW and OW variation observed in the radiata pine branches were mostly in agreement with previous data derived from bent trunks of conifer species [2]. Although gravity stress has little impact on tracheid wall expansion in radial and tangential directions (Figure 1), it does affect longitudinal growth of tracheids as CW has longer tracheids than OW [2].

Differential gene transcription could provide molecular evidence for the drastic variation between CW and OW. The xylem transcriptome changes in CW and OW of radiata pine branches (28-29%) are among the highest in a number of microarray comparisons with regard to radiata pine wood development, including earlywood vs. latewood (11-30%) [22], juvenile wood vs. mature wood (9.2-19.3%) [23], high stiffness vs. low stiffness wood (3.4-14.5%) [24], high density vs. low density wood (10-19%) [25]. Genes differentially transcribed in CW and OW had various functions in cell division, cell expansion, primary wall synthesis, secondary wall deposition, hormone and calcium signallings, transcription and environmental stresses. The extensive transcriptome remodelling and divergent functions of differentially transcribed genes could underlie drastic CW and OW variation observed in radiata pine branches. Genes involved in cell wall formation, hormone and calcium signallings, and various environmental stresses were mostly up-regulated in CW. Thus, CW experienced more transcriptome remodelling than OW, resulting in greater phenotypic variation between CW and wood formed in normal conditions (NW) compared to that between OW and NW [2].

### Cytoskeleton-related genes affect cellulose microfibril orientation

The cytoskeleton is made up of microtubules, actin filaments, and intermediate filaments [26]. There is growing evidence that cortical microtubules play a key role during the crystallization of cellulose microfibrils [27] by directing their orientation in the wall [28]. Up-regulation of *alpha-* and *beta*-*tubulins* in CW (with larger MFA) of radiata pine branches is in agreement with previous study using bent trunks of maritime pine [16]. Association of allelic variation in an *alpha-tubulin* with MFA was observed in secondary xylem of loblolly pine [29]. In angiosperms, several *alpha-* and *beta*-*tubulins* were highly transcribed in TW (with reduced MFA) formed in bent poplar trunks [30] and eucalypt branches [17]. Over-transcription of an eucalypt *beta*-*tubulin* gene in transgenic xylem directly influenced MFA [31]. Taken together, the functions of *tubulin* genes involved in cellulose microfibril orientation of secondary xylem have been conserved in both gymnosperms and angiosperms.

Actin filaments are much less rigid compared to microtubules [32]. Interaction of actin filaments with cortical microtubules altered the orientation of cellulose microfibrils in cultured cotton fiber cells [33]. Our study identified two *ADFs* (*ADF* and *ADF-like*) that were exclusively up-regulated in CW of branches. ADF plays an important role in regulating the optimum balance between unpolymerised actin molecules and assembled actin filaments [34]. Genes involved in actin filaments showed different transcription patterns in reaction wood between branches (this study) and bent trunks [16] in conifers. For example, *actin polymerizing factors* up-regulated in CW of bent trunks in spring were not identified in CW of branches in either spring or autumn, suggesting their responses exclusive to bending forces rather than gravity stimulus. In contrast, genes (i.e., four *actins*, two *ADFs* and two *actin bundling proteins*) with differential transcription in branches were not identified in bent trunks, highlighting their possible roles in response to gravity stress.

### Secondary cell wall genes confer tracheid wall thickness and wood density

This study identified many secondary cell wall genes with preferential transcription in CW of radiata pine branches (Table 3 and 4). Three *PrCesA* genes (*PrCesA3*, *7*, *11*) up-regulated in CW were previously clustered as secondary wall genes and *PrCesA10* preferentially transcribed in OW is a primary wall gene [35]. Several *CesAs* were also up-regulated in CW of bent maritime pine [16], TW of bent eucalypts [36, 37] and poplars [38, 39]. Besides, *CesA-like* and *SuSy* genes were up-regulated in CW of both branches (this study) and bent trunks of conifers [16]. In Scots pine SuSy activity was observed to peak in the zone of maturing tracheids where the secondary wall is formed, and its transcription was lower in primary wall tissues [40]. Over-transcription of a *SuSy* gene increased cellulose content, secondary wall thickness and wood density in poplars [41].

Lignin biosynthesis consists of three major steps: shikimate pathway, monolignol pathway and monolignol polymerization [42]. Phenylalanine is an end product of the shikimate pathway with seven enzymes involved [43]. Five of these genes were up-regulated in CW of radiata pine branches, including *shikimate kinase 2*, *chorismate synthase*, *chorismate mutase*, *3-deoxy-D-arabino-heptulosonate 7-phosphate synthase* (*DAHPS*) and *5-enolpyruvylshikimate 3-phosphate synthase* (*EPSPS*) (Additional file 1). Phenylalanine and other precursors for monolignol biosynthesis are extensively methylated in the S-adenosyl methionine (SAM) dependent reaction [44]. Genes related to SAM metabolism (*SAMS*, *SAMDC*, *MetE* and *methionine synthases*) were up-regulated in CW of radiata pine branches. Preferential transcription of genes related to shikimate pathway and SAM metabolism in CW may result in more production of monolignol precursors. Finally, monolignol synthesis could be also enhanced in CW due to up-regulation of several key genes involved in monolignol pathway (e.g., *PAL*, *C4H*, *COMT*, *4CL* and *CCR-like*). Most of these lignin-related genes were also up-regulated in CW of bent trunks in maritime pine [16] and TW of bent eucalypts [36]. Besides, several other lignin-related proteins were highly presented in CW of bent trunks in maritime pine (COMT, caffeoyl CoA-O-methyltransferase and SAMS) [45] and in Japanese cypress at the transcript level (*laccases*, *COMT* and *methionine synthase*) [13]. In summary, up-regulation of lignin-related genes in CW provided the molecular basis for its higher lignin content and thicker tracheid walls compared to that in OW.

A number of cell wall structural protein genes (*AGPs, AGP-like*, *glycine-rich proteins and proline-rich proteins*) were exclusively up-regulated in CW of radiata pine branches. In loblolly pine six *PtaAGPs* were predominantly transcribed in secondary xylem development [46]. Our study revealed that *FLAs* were differentially transcribed in either CW (*FLA1* and *8*) or OW (*FLA10*, *17* and *26*) of radiata pine branches. In angiosperms *FLAs* were up-regulated in TW of bent poplar trees [47] and eucalypt branches [17]. Gene function studies further confirmed that *FLAs* affect MFA and tensile stiffness in transgenic eucalypts and Arabidopsis by altering cellulose deposition and the integrity of the cell wall matrix [48].

Tracheid wall thickness and wood density are determined by secondary cell wall synthesis and deposition. Up-regulation of genes related to cellulose and lignin biosynthesis and cell wall structure in CW of radiata pine branches coincided with its drastically increased tracheid walls and wood density. These results were generally in agreement with previous studies in the bent trunks of maritime pine [16] and eucalypts [36] as well as in radiata pine juvenile wood with higher density [25]. However, it has been well documented that CW has lower cellulose content [2, 11]. This is because lignin synthesis is also greatly increased in CW as suggested in this study and demonstrated elsewhere [2]. Thus, CW has relatively lower cellulose content.

### Genes involved in cell division and primary wall modification implicate wood growth and tracheid dimensions

The quantity of wood formation is largely related to cell division and expansion during primary cell wall development. Four cell division-related genes were identified with up-regulation in CW of radiata pine branches. Rapid cell division in CW could be an earlier response of reaction wood formation in conifer branches under gravity stress. It can partly explain the greater wood growth on the lower side of branches.

Tracheids are structures of three dimensions (radial, tangential and longitudinal directions) which are determined in cell expansion during primary wall formation. Several *expansins* and *XETs* were differentially transcribed in CW and OW of radiata pine branches (this study) and bent trunks of maritime pine [16]. *XETs* can cut and rejoin xyloglucan (XG) chains, and are believed to be important regulators of primary wall expansion [49]. Different genes involved in pectin biosynthesis were up-regulated in CW (*pectin lyase-like*) or OW (*pectin lyase 2*, *pectinesterase-like* and *pectin-glucuronyltransferase*) of radiata pine branches. Differential transcription of these genes in CW and OW could provide molecular evidence for their similar tracheid diameters (either radial or tangential directions). On the other hand, a gene encoding ovule/fiber cell elongation protein with up-regulation in OW could suggest its possible function in the longitudinal growth of tracheids.

### Gravity stress triggers hormone, calcium and other environmental signals

Plant gravitropism is a complex process including three major stages: gravity perception, signal transduction, and growth response. In this study many genes related to hormone and calcium signalling as well as environmental stresses were up-regulated in CW of branches; while only a few genes in these categories were preferentially transcribed in OW (Table 5 and 6). These results provided valuable clues for the understanding of reaction wood formation in response to gravity stimulus during earlier perception and signal transduction.

Some hormone signalling genes with up-regulation in CW of radiata pine branches (Table 5) had preferential transcription in CW of inclined pines [14, 45] and TW of bent eucalypts [36]. Auxin is widely believed to be the primary effector of gravitropism since its asymmetric distribution drives the gravitropic growth [50]. Gravity stimulus also induces other hormones, such as ethylene (on the lower side of branches [51] and bent trunks [52]) and gibberellins (TW in tilted *Acacia mangium* seedlings [53]). Cytokinin increased secondary xylem formation with higher lignification and thicker cell walls [54]. The identified hormone signalling genes (Table 5) and other TF genes (Additional file 2) could provide additional candidates of gravity preceptors or signal transduction.

Calcium (Ca^2+^) signalling has a strong relationship with plant gravitropism [55]. It has functions in all steps of the signal transduction pathway by acting as a second messenger to mediate auxin redistribution [55]. A *calcium/calmodulin-dependent protein kinase* from maize showed light-regulated gravitropism [56]. Calcium has been proven a role in secondary xylem development and CW formation [57]. In the present study several calcium signalling genes were consistently up-regulated in CW of branches in both spring and autumn (Table 5), providing further evidence for calcium signals with roles in reaction wood formation. The identified calcium signalling genes could be important candidates of earlier signals of conifer reaction wood formation in response to gravity stress.

The majority of differentially transcribed genes involved in various environmental stresses (e.g., water, light, diseases and salt) were up-regulated in CW of branches (Table 6). This is because rapid CW formation requires more resources for cell division, primary wall formation and secondary wall deposition that trigger different environmental stresses. Up-regulation of three light signalling genes (*light-inducible protein ATLS1*, *light-induced protein-like* and *phytochrome*) in CW of radiata pine branches could be a result of reduced light radiation on the lower side of branches. Phytochromes are red and far-red light photoreceptors, and they regulate a large number of genes involved in hormone signalling or enzymes involved in cell wall modification [58]. In hypocotyls gravitropism phytochromes inhibited four phytochrome-interacting factors [59]. Thus, plant gravitropism may be regulated by the interaction between light, hormone and calcium signallings [60, 61].

## Conclusions

Compression wood formed in radiata pine branches showed greater radial growth, thicker tracheid walls, larger microfibril angle (MFA), higher density and lower stiffness, but similar tracheid diameters compared to its opposite wood. Extensive remodelling of the xylem transcriptomes (29%) observed in compression and opposite wood could provide molecular evidence for their drastic variation in tracheid and wood traits. Many genes involved in cell division, cellulose biosynthesis, lignin pathway and microtubules were exclusively up-regulated in compression wood, conferring its greater radial growth, thicker tracheid walls, higher density, larger MFA and lower stiffness. In contrast, genes related to cell expansion and primary wall modification were differentially transcribed in either compression or opposite wood, implicating their similar tracheid diameters but different tracheid lengths. Of particular interest, a broad range of genes related to hormone and calcium signalling and various environmental stresses were exclusively up-regulated in compression wood, suggesting possible earlier molecular signatures of plant gravitropism during reaction wood formation in conifers. The first transcriptome profiling of radiate pine branches provides more accurate insights into the molecular basis of reaction wood formation in response to gravity stimulus without external bending forces.

## Methods

### Plant materials and sampling

Six trees with well-developed branches were selected from a radiata pine commercial plantation located at Bondo, NSW, Australia (35º 16' 44.04" S, 148º 26' 54.66" E). These trees were originated from seedlings with different genotypes and they were 13 years old at the time of sampling. The largest branch from each tree was further selected for study, including three branches sampled in autumn and three sampled in spring. Bark was removed from the base part (about 10 cm in length) of each branch. Developing xylem tissues were scraped from the exposed upper and lower side surface respectively with a sharp chisel. Samples were immediately placed into 50 ml BD Falcon™ tubes filled with liquid nitrogen. One branch disc (approximately 5 cm in length) was then cut off from the larger end of each branch adjacent to the base part used for developing xylem sampling. Location of upper and lower side zones was immediately marked on all discs collected from branches.

### Measurements of tracheid and wood traits

After removing the bark from each branch disc a block of wood (about 2 cm in length in both tangential and longitudinal directions) was cut from the top of upper side to the bottom of lower side through pith. A twin-blade saw was used to trim the wood blocks to produce strips (containing pith) of 2 mm in the tangential direction and 7 mm in the longitudinal direction. The wood strips were characterized using the SilviScan® instrument [18, 19]. A total of eight tracheid and wood traits were measured, including tracheid wall thickness, radial diameter, tangential diameter, coarseness (tracheid mass per unit length), specific surface (tracheid surface area per unit mass), cellulose microfibril angle (MFA, the angle of cellulose fibrils in wood cell walls versus the longitudinal cell axis), as well as wood density (the dry weight per unit volume of wood) and stiffness or modulus of elasticity (MOE) (the degree of wood deflected when a load is applied perpendicular to the grain). All eight traits were analyzed at 25 μm interval across the wood strips.

### Microarray experiments and data analysis

Total RNA was extracted from developing xylem tissues using a modified CTAB method [62]. Transcript abundance on the upper and lower side of branches sampled in spring and autumn was compared, respectively, using radiata pine cDNA microarrays containing 18,432 clones derived from six developing xylem libraries [22, 35]. Of these cDNAs, 6,169 were randomly sequenced and assembled into 3,320 xylem unigenes (986 contigs and 2,334 singletons) [22, 35]. A dye swap was performed for each biological replicate, resulting in a total of six replicates in each of the two microarray experiments.

Construction of cDNA microarrays, synthesis of probes and microarray hybridization were performed in methods described previously [22–24]. Hybridized microarrays were scanned using a GenePix Personal 4100A scanner (Axon Instruments, CA). Images were pre-processed using GenePix® Pro 6.0 (Axon Instruments, CA). Median values of fluorescence intensity of the red and green colours were used to generate a ratio representing the difference of gene transcription in the two tissues being compared. Differential gene transcription in the six microarrays of each experiment were jointly normalized at both print-tip and slide scale levels using GEPAS v3.1 [63]. The raw dataset of all 12 microarrays was registered in the NCBI GEO database with accession number GSE47167. Mean fold changes of gene transcription in CW compared to OW ≥ 1.5 times (or log-2 ratio ≥ 0.584 and ≤ -0.584) and P-values ≤ 0.05 calculated with Cyber-T [64] were used as thresholds for the selection of differentially transcribed unigenes. Putative candidate genes were further shortlisted after removing redundant unigenes showing identical accession numbers in the UniProt known proteins and TIGR gene indices databases.

### Validation of microarray gene transcription

Microarray results of selected genes were validated using the reverse transcriptase-multiplex ligation dependent probe amplification (RT-MLPA) method [65]. A total of seven genes consistently up-regulated in CW or OW in both spring and autumn were selected for validation, including four genes for CW: *cellulose synthase 3* (*PrCesA3*), *PrCesA11*, *cinnamic acid 4-hydroxylase* (*C4H*) and *plastocyanin-like* (*PCL*); and three genes for OW: *peroxidase* (*PER*), *E3 ubiquitin protein ligase* (*UPL1*) and *retinoblastoma-like protein* (*RBL*) (Additional file 1). Developing xylem (CW and OW) sampled in autumn for the microarray experiment was used in the validation, including three biological and four technical replicates. Mean log-2 ratios of the 12 replicates were calculated for selected genes and then compared with microarray results.

Approximately 400 ng of purified total RNA was reverse transcribed into first strand cDNA using the ImProm-II Reverse Transcription System (Promega, WI). The cDNA was hybridized at 60°C overnight with bulked RPO (right probe oligo) and LPO (left probe oligo) probes designed for the selected genes (Additional file 3). Ligation and PCR amplification were performed with SALSA D4 primer. Individual gene fragments were separated from the mixed PCR products using a CEQ™ 8000 Genetic Analysis System (Beckman Coulter, CA) and relative gene transcription levels were determined using the built-in software.

## Electronic supplementary material

Additional file 1: **Genes differentially transcribed in compression (CW) and opposite wood (OW) of radiata pine branches sampled in spring and autumn.** In total, 846 genes were identified with differential transcription in CW (693) and OW (153) of radiata pine branches sampled in spring; while 872 genes were preferentially transcribed in CW (511) and OW (361) sampled in autumn. (XLS 506 KB)

Additional file 2: **Transcription factors differentially transcribed in compression (CW) and opposite wood (OW) of radiata pine branches.** (XLS 25 KB)

Additional file 3: **LPO (left probe oligo) and RPO (right probe oligo) of selected differentially transcribed genes involved in the validation.** A total of seven differentially transcribed genes identified in the microarray experiments were selected in the validation by reverse transcriptase-multiplex ligation dependent probe amplification (RT-MLPA). Their LPO and RPO sequences were listed in the table. (XLS 24 KB)
